# Genetic Alterations in Intervertebral Disc Disease

**DOI:** 10.3389/fsurg.2016.00059

**Published:** 2016-11-21

**Authors:** Nikolay L. Martirosyan, Arpan A. Patel, Alessandro Carotenuto, M. Yashar S. Kalani, Evgenii Belykh, Corey T. Walker, Mark C. Preul, Nicholas Theodore

**Affiliations:** ^1^Department of Neurosurgery, St. Joseph’s Hospital and Medical Center, Barrow Neurological Institute, Phoenix, AZ, USA; ^2^Division of Neurosurgery, College of Medicine, University of Arizona, Tucson, AZ, USA; ^3^College of Medicine – Phoenix, University of Arizona, Phoenix, AZ, USA; ^4^Laboratory of Neurosurgery, Irkutsk Scientific Center of Surgery and Traumatology, Irkutsk, Russia; ^5^Irkutsk State Medical University, Irkutsk, Russia

**Keywords:** back pain, biomarker, degeneration, disc, gene expression, herniation, personalized care, single-nucleotide polymorphism

## Abstract

**Background:**

Intervertebral disc degeneration (IVDD) is considered a multifactorial disease that is influenced by both environmental and genetic factors. The last two decades of research strongly demonstrate that genetic factors contribute about 75% of the IVDD etiology. Recent total genome sequencing studies have shed light on the various single-nucleotide polymorphisms (SNPs) that are associated with IVDD.

**Aim:**

This review presents comprehensive and updated information about the diversity of genetic factors in the inflammatory, degradative, homeostatic, and structural systems involved in the IVDD. An organized collection of information is provided regarding genetic polymorphisms that have been identified to influence the risk of developing IVDD. Understanding the proteins and signaling systems involved in IVDD can lead to improved understanding and targeting of therapeutics.

**Materials and methods:**

An electronic literature search was performed using the National Library of Medicine for publications using the keywords genetics of IVDD, lumbar disc degeneration, degenerative disc disease, polymorphisms, SNPs, and disc disease. The articles were then screened based on inclusion criteria that included topics that covered the correlation of SNPs with developing IVDD. Sixty-five articles were identified as containing relevant information. Articles were excluded if they investigated lower back pain or just disc herniation without an analysis of disc degeneration. This study focuses on the chronic degeneration of IVDs.

**Results:**

Various genes were identified to contain SNPs that influenced the risk of developing IVDD. Among these are genes contributing to structural proteins, such as *COL1A1, COL9A3, COL9A3, COL11A1*, and *COL11A2, ACAN*, and *CHST3*. Furthermore, various SNPs found in the vitamin-D receptor gene are also associated with IVDD. SNPs related to inflammatory cytokine imbalance are associated with IVDD, although some effects are limited by sex and certain populations. SNPs in genes that code for extracellular matrix-degrading enzymes, such as MMP-1, MMP-2, MMP-3, MMP-9, MMP-14, ADAMTS-4, and ADAMTS-5 are also associated with IVDD. Apoptosis-mediating genes, such as caspase 9 gene (*CASP9*), *TRAIL*, and death receptor 4 (*DR4*), as well as those for growth factors, such as growth differentiation factor 5 and VEGF, are identified to have polymorphisms that influence the risk of developing IVDD.

**Conclusion:**

Within the last 10 years, countless new SNPs have been identified in genes previously unknown to be associated with IVDD. Furthermore, the last decade has also revealed new SNPs identified in genes already known to be involved with increased risk of developing IVDD. Improved understanding of the numerous genetic variants behind various pathophysiological elements of IVDD could help advance personalized care and pharmacotherapeutic strategies for patients suffering from IVDD in the future.

## Introduction

Over 80% of all people will experience some form of lower back pain in their lifetime (1–3). Symptomatic intervertebral disc (IVD) degeneration (IVDD) is a common cause of lower back pain, yet the etiology and pathophysiology underlying IVDD remain poorly understood (4, 5). Although various environmental factors such as smoking, age, gender, and mechanical load increase the risk of IVDD, it is hypothesized that up to 74% of the etiology of IVDD is due to heritability (2, 6). With lower back pain costing over $100 billion/year in the United States, it is essential to investigate both the environmental and genetic predispositions to IVDD (5).

**Figure 1:**
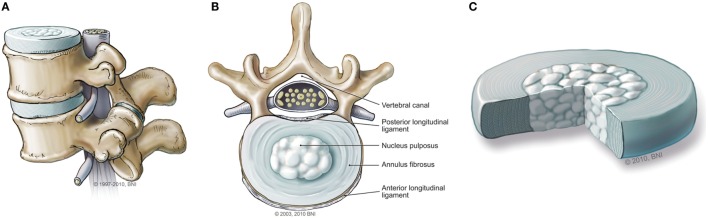
**(A)** Normal intervertebral disc (IVD) from the sagittal view. **(B)** Normal IVD from the axial view. **(C)** Magnified illustration of an IVD. Used with permission from Barrow Neurological Institute, Phoenix, AZ, USA.

The normal IVD is composed of two parts: the outer annulus fibrosis (AF) region and the central nucleus pulposus (NP) (Figure 1). The AF consists of fibroblast-like cells with elongated nuclei placed between concentric layers of collagen fibers (3). The extracellular matrix (ECM) of the AF can be described as a fibrocartilaginous structure consisting of predominantly collagen-I fibers (60% of total dry weight), with low proteoglycan content (25%) and low water retention (5, 7). Its primary function is to provide structural integrity to the disc and hold the contents of the NP in the center (3, 5). The NP is a gelatinous structure with chondrocyte-like cells that secrete collagen II. Dispersed throughout the collagen fibers are an abundance of proteoglycans, predominantly aggrecan, which are responsible for facilitating water retention (3, 5, 8). The primary function of the NP is to create hydrostatic pressure to resist axial compression (5, 7).

Intervertebral disc degeneration seems to be an irreversible process that can begin as early as the second decade (5). The first molecular change that occurs at the beginning of degeneration is a reduced ability of the NP to retain water and consequently maintain a significant hydrostatic pressure (7). These changes result in decreased disc height and reduced ability of the spine to withstand compression. Over time, the collagen fibers and other ECM components of both the NP and AF are degraded and reduced in quantity (8). Upregulation of degradative systems such as apoptosis, inflammation, and matrix metalloproteinases (MMPs) further damage the existing (9–13). The past 20 years of genomic research has revealed an astounding number of genetic polymorphisms of various genes that are correlated with increased risk of developing IVDD. Polymorphisms in the genes coding for collagen, aggrecan, interleukins (ILs), apoptosis factors, vitamin D receptor (VDR), MMPs, and other critical proteins involved in IVDD are examined in this paper. Although previous reviews have documented the various single-nucleotide polymorphisms (SNPs) that are associated with IVDD, we aim to provide an up-to-date and comprehensive review of the subject (5, 7, 8).

With an improved understanding of the genetic variants associated with IVDD, we hope to help advance personalized care and pharmaceutical therapies for patients suffering from IVDD. Across various medical specialties, genome sequencing has begun to play a significant role in improving the care provided to patients (14). Human genome analysis allows physicians to obtain a deeper understanding of the pathophysiology of diseases to provide improved risk and prognostic assessments to patients. Furthermore, information regarding genetic variants provides insight into therapeutic options as physicians are better able to target the underlying disease-causing mechanisms (15). Throughout this paper, we will explore the complexity and diversity of the molecular and genetic factors involved in IVDD. Genetic variants from various molecular pathways are investigated including inflammatory, degradative, homeostatic, and structural systems. Clinical use of genome analysis allows physicians to pinpoint which systems and particular pathways are involved with the patient’s unique case of IVDD and subsequently provide personalized and improved health care.

## Methods

An electronic literature search was performed using the National Library of Medicine for publications using these keywords: genetics of IVDD, lumbar disc degeneration, degenerative disc disease, polymorphisms, SNPs, and disc disease. The articles were then screened based on inclusion criteria that included topics that covered the correlation of SNPs with developing IVDD. Furthermore, articles containing supporting information regarding the treatment and diagnosis of IVDD were included. Sixty-five total articles were identified as containing relevant information. Articles were excluded if they investigated lower back pain or disc herniation without an analysis of disc degeneration or study of correlation with SNPs. This investigation focuses on the chronic degeneration of IVDs and the genetic factors that influence its development.

## Treatment for IVDD

**Figure 2:**
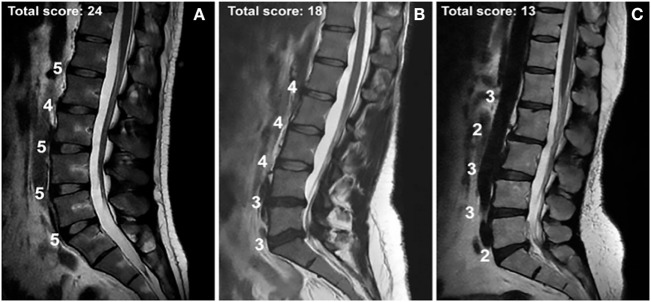
**MRIs of intervertebral disc disease in patients with total Pfirrmann scores of (A) 24, (B) 18, and (C) 13 and the assessed score for each lumbar disc**. The Pfirrmann grading scale for disc degeneration classifies discs into 5 grades according to the amount of degeneration. Grade 1 corresponds to a hyperintense healthy disc, while grade 5 corresponds to a hypointense severely degenerated disc. The figure contains a point system corresponding to each intervertebral disc from L1 to S1. Five points were given for a grade 1 score, four points for a grade 2, three for a grade 3, two for a grade 4, and one for a grade 5. The highest possible total score is 25; the lowest possible score is 5. Used with permission from Toktas et al. (20).

Diagnosis of IVDD requires a careful history, physical examination, and, most importantly for the experimental studies included in this review, magnetic resonance imaging (MRI) of the spine. The majority of studies that were included in this literature review used axial and/or sagittal T2-weighted MRIs to evaluate the lumbar spine of the patients (Figure 2). Once the patient has been accurately diagnosed with disc degeneration, limited approved therapeutics are available to abate the progression of the degeneration. Therapy to combat IVDD and the associated degeneration and pain is highly complex, and it can be difficult to predict its effectiveness. Recently, researchers have found success utilizing targeted molecular and gene therapies in an attempt to mitigate degradation and even promote anabolic processes. Injection of recombinant human bone morphogenetic protein 7 (BMP-7, also known as osteogenic protein 1, OP-1) has been successful in a rabbit model (16). BMP-7 injection restored the disc height and biomechanical properties of the damaged disc. Other growth factors such as rhGDF-5 have also shown great promise (17). In that study, a single injection was shown to increase disc height. Furthermore, rhGDF-5 injection has been shown to reduce the expression of ADAMTS-4 and ADAMTS-5 proteins for which, the genes have been identified to contain SNPs associated with altered risk of developing IVDD (18). This serves as an excellent example of the intersection of providing targeted therapy and gene analysis of patients with IVDD. RhGDF-5 injections may serve as the most effective therapy in a patient who has been screened for having high-risk IVDD due to SNPs in their ADAMTS-4 and -5 genes (18). Furthermore, injection of other molecules, such as TGF-β1 and BMP-2, has been shown to inhibit MMP-1 expression and increase expression of aggrecan protein. Genes for both MMP-1 and aggrecan protein are known to contain SNPs that predispose patients to develop IVDD (19). Combining the specific effects of these anabolic therapies with an understanding of the individualized molecular profile of each patient may yield a highly effective treatment. Therefore, it is essential that research efforts continue to progress in both targeted therapies and gene analysis of IVDD.

The most effective experimental approach in the treatment of IVDD is the use of viral vectors in gene therapy. *In vitro* bovine experimentation with the delivery of sex-determining region Y box 9 (*SOX9*) and *BMP7* through an adenovirus vector revealed increased expression of type II collagen and an increase in disc height (21). Another experiment showed that cells virally transduced with Ad-BMP-4 and -14 displayed an increase in collagen deposition, whereas cells transduced with Ad-BMP-2 and -7 displayed an increase in proteoglycan accumulation (19). The consistently positive results obtained from these experiments suggest a largely uncharted frontier exists in the use of personalized medicine for IVDD.

## Grading IVDD

Physicians utilize various grading systems to assist in diagnosing and measuring the severity of IVDD and to determine the most standardized and objective classification of disc degeneration. A popular and widely accepted scale is the Pfirrmann grading system. The system includes grades 1–5, where grade 1 signifies a normal disc with homogenous hyperintensity on MRI, and grade 5 signifies a collapsed disc space with a hypointense signal (Figure 2) (22). A common critique of the system is its subjectivity. It is often modified or combined with other grading systems such as Modic changes to create an objective, reproducible system (23). Some physicians and research groups opt to develop their own grading system, while others utilize classification systems such the one developed by Schneiderman et al. (13, 24–26). Once a patient’s disc degeneration is objectively graded, a standard of care can be established.

## Gene Polymorphisms Associated with IVDD

Table 1 (1, 4, 11–13, 20, 24, 25, 27–57) presents comprehensive information on the research studies that have investigated genes with SNPs associated with IVDD and their protein products. Table 2 (1, 4, 11–13, 20, 24, 25, 27–58) summarizes the protein systems associated with such changes in the respective genes.

**Table 1:** **Summary of research studies on single-nucleotide polymorphisms (SNPs) associated with intervertebral disc degeneration (IVDD)**.

Reference	Protein	SNP	Study population	Results
Pluijm et al. (27)	Collagen I	COL1A1 Sp1	966 Elderly Dutch subjects (>65 years old)	TT genotype odds ratio (OR) = 3.6 compared with GT or GG
Tilkeridis et al. (28)	Collagen I	COL1A1 Sp1	24 Greek military recruits (mean age 29 years old), 12 controls (mean age 25 years old)	TT genotype found in 33.3% of patients with IVDD and 0% of controls; GT genotype found in 66.7% of patients with IVDD and 41.7% of controls
Toktas et al. (20)	Collagen I	COL1A1 Sp1	75 Southern European men with IVDD, 25 controls (35–45 years old)	T allele associated with more severe IVDD based on Pfirrmann scores
Annunen et al. (29)	Collagen IX	COL9A2 Trp2	157 Finnish subjects (19–78 years old) with sciatic pain, 174 controls	Trp2 allele OR = 4.5 compared with patients without allele
Kales et al. (30)	Collagen IX	COL9A2 Trp2	105 Greek patients with IVDD, 102 controls (<60 years old)	No association between Trp2 and IVDD
Toktas et al. (20)	Collagen IX	COL9A2	75 Southern European men with IVDD, 25 controls (35–45 years old)	Did not find association between Trp2 and IVDD
Zhang et al. (31)	Collagen IX	COL9A2 rs12077871, rs12722877, rs7533552	Meta-analysis with 1522 lumbar disc disease (LDD) cases, 1646 controls	No association between the SNPs and IVDD
Paassilta et al. (32)	Collagen IX	COL9A3 Trp3	171 Finnish patients with sciatic pain, 321 controls (mean age 45 years old)	Trp3 allele OR = 2.7 for developing IVDD
Solovieva et al. (33)	Collagen IX	COL9A3 Trp3	135 Finnish male patients (40–45 years old)	Trp3 allele OR = 7.0 for developing dark nucleus pulposus; OR = 8.0 for degeneration of spine in absence of IL-1 βT3954 SNP allele
Toktas et al. (20)	Collagen IX	COL9A3 Trp3	75 Southern European men with IVDD, 25 controls (35–45 years old)	Trp3 allele associated with more severe degeneration based on Pfirrmann scores
Solovieva et al. (33)	Collagen XI	COL11A2 G to A SNP within intron 9	135 Finnish male patients (40–45 years old)	Risk allele OR = 2.1 for increased risk of disc bulges
Videman et al. (4)	Collagen XI	rs2072915, rs9277933, rs2076311, rs1337185, rs1463035	588 Finnish male twins (35–70 years old)	Some SNPs were significantly associated with reduced disc signal on MRI while others were associated with disc bulging
Rajasekaran et al. (34)	Collagen XI	rs1337185	308 Indian male patients with mild Total Disc Degenerative Score (mean age 29.6 years old), 387 Indian male patients with severe TDDS (mean age 31.7 years old)	SNP rs1337185 OR = 1.55 for developing IVDD
Virtanen et al. (35)	Interleukin-1a	-889C/T	150 Finnish men (38–56 years old), 61 control subjects	TT genotype OR = 7.87 for developing IVDD compared with patients with CC genotype
Eskola et al. (36)	Interleukin-1a	-889C/T	96 Danish adolescents with IVDD, 57 controls (mean age 13.1 years old at the beginning of the study)	In girls, the T-allele OR = 2.82 for disc degeneration
Noponen-Hietala et al. (37)	Interleukin-6	T15A within exon 5	155 Finnish subjects (17–78 years old), 179 controls (20–69 years old)	AA or AT genotypes OR = 4.4 for IVDD
Eskola et al. (13)	Interleukin-6	rs1800796, 572G/C	66 Children with LDD, 154 controls; total 352 children studied (mean age 13.1 years old at the beginning of the study)	C allele OR = 6.71 for IVDD in females
Eskola et al. (36)	Interleukin-6	rs1800797(Risk allele G), rs1800795 (Risk allele G)	96 Danish adolescents with IVDD, 57 controls (mean age 13.1 years old at the beginning of the study)	GA genotype of rs1800797 OR = 0.27 for IVDD; GC genotype of rs1800895 OR = 0.26 for IVDD in males
Dong et al. (38)	Matrix metalloproteinase (MMP)-2	-1306C/T	162 Chinese young adults with IVDD (mean age 25.4 years old), 318 controls (mean age 24.1 years old)	CC genotype OR = 3.08 for developing IVDD; CC genotype also associated with more severe forms of IVDD
Zhang et al. (39)	MMP-2	-735C/T	1008 Chinese Han patients with LDD (mean age 50.12 years old), 906 controls (mean age 49.54 years old)	Patients with TT or CT genotype OR = 0.413 for developing IVDD. CC genotype OR = 2.5 for developing IVDD compared with TT
Sun et al. (11)	MMP-9	-1562C/T	408 Northern Chinese young adults with IVDD (18–21 years old), 451 controls (16–30 years old)	TT and CT genotypes OR = 2.14 for developing IVDD
Takahashi et al. (40)	MMP-3	5A Variant	54 Young Japanese (18–28 years old) and 49 elderly (64–94 years old) patients	5A/6A and 5A/5A genotypes associated with increased risk of IVDD in elderly
Yuan et al. (12)	MMP-3	5A Variant	178 Chinese patients with IVDD (mean age 48.5 years old), 284 controls (mean age 40.6 years old)	5A allele OR = 2.5 for developing IVDD; 5A allele also associated with more severe forms of IVDD
Zhang et al. (41)	MMP-14	-378T/C	908 Chinese Han IVDD patients with IVDD (mean age 51.12 years old), 906 controls (mean age 51.54 years old)	TT genotype OR = 1.59 for developing IVDD compared with CC genotype
Liu et al. (42)	A disintegrin and metalloproteinase with thrombospondin motif (ADAMTS)-4	rs4233367: 1877C/T	482 Chinese Han patients (mean age 42.6 years old), 496 controls (mean age 41.4 years old)	TT genotype OR = 0.21 for developing IVDD compared with CC genotype
Rajasekaran et al. (34)	ADAMTS-5	rs162509	308 Indian male patients with mild Total Disc Degenerative Score (mean age 29.6 years old), 387 Indian male patients with severe TDDS (mean age 31.7 years old)	Risk allele OR = 1.281 for developing IVDD
Kawaguchi et al. (43)	Aggrecan	VNTR	64 Young women (20–29 years old), 32 cases, 32 controls	Patients with 18 or 21 repeats were at greater risk of developing IVDD than patients with longer alleles
Eser et al. (24)	Aggrecan	VNTR	150 Turkish young adults with IVDD, 150 controls (20–30 years old)	A13–26 length alleles associated with higher risk of IVDD than longer alleles
Xu et al. (44)	Aggrecan	VNTR	Meta-analysis	Repeats of <25 OR = 1.85 for developing IVDD
Gu et al. (45)	Aggrecan	VNTR	Meta-analysis with 965 cases and 982 controls	A13–25 repeats OR = 1.52 for developing IVDD. In Asian patients specifically, OR = 1.65
Solovieva et al. (46)	Aggrecan	VNTR	132 Finnish middle-aged men (41–46 years old)	A26 allele associated with increased risk of dark NP on MRI. A26/A26 genotype OR = 2.77 for dark NP compared with longer or shorter alleles
Song et al. (47)	Carbohydrate sulfotransferase 3 (CHST3)	rs4148941	4043 Patients with LDD; 28,599 controls	AA or AC genotype OR = 1.49 for developing IVDD
Videman et al. (48)	Vitamin D receptor	FokI	85 Pairs of Finnish twins (35–69 years old)	Ff and ff genotypes associated with reduced disc signal intensity on MRI
Eser et al. (24)	Vitamin D receptor	FokI	150 Turkish young adults with IVDD, 150 controls (20–30 years old)	ff Genotype associated with more severe grades of IVDD (grades III, IV)
Vieira et al. (49)	Vitamin D receptor	FokI	121 Brazilian patients with IVDD (mean male age 46.0 years old, female 45.2 years old), 131 Brazilian population controls (mean male age 33.8 years old, female 33.9 years old)	T allele OR = 1.58 for developing IVDD. Ff and ff genotypes OR = 1.742 for developing IVDD in Hispanics; OR = 1.293 in Asians
Videman et al. (48)	Vitamin D receptor	TaqI	85 Pairs of Finnish twins (35–69 years old)	tt Genotype associated with reduced disc signal intensity on MRI
Kawaguchi et al. (50)	Vitamin D receptor	TaqI	205 Japanese young adults (mean age 22)	Tt genotype associated with multilevel disc degeneration
Eser et al. (24)	Vitamin D receptor	TaqI	150 Turkish young adults with IVDD, 150 controls (20–30 years old)	TT genotype associated with milder forms of IVDD compared with tt genotype
Toktas et al. (20)	Vitamin D receptor	TaqI	75 Southern European men with IVDD, 25 controls (35–45 years old)	tt Genotype associated with more severe forms of IVDD based on Pfirrmann scores
Yuan et al. (12)	Vitamin D receptor	ApaI	178 Chinese patients with IVDD (mean age 48.5 years old), 284 controls (mean age 40.6 years old)	Risk allele OR = 1.70 for developing IVDD
Zawilla et al. (51)	Vitamin D receptor	ApaI	84 Egyptian patients with IVDD (mean age 44.2 years old) and 60 controls (mean age 43.3 years old)	Mutant T allele OR = 3.1 for developing IVDD; T allele also associated with more severe forms of IVDD
Guo et al. (1)	Caspase-9	rs4645978: -1262A/G	154 Patients with LDD (20–65 years old), 216 controls (20–65 years old)	GG genotype of rs4645978 OR = 2.76 for developing IVDD compared with AA genotype
Mu et al. (52)	Caspase-9	rs4645978: -1262A/G	892 Chinese male soldiers: 305 cases (mean age 21.94 years old), 587 controls (mean age 22.09 years old)	G allele OR = 2.059 for developing IVDD
Xu et al. (53)	TNF (tumor necrosis factor)-related apoptosis-inducing ligand (TRAIL)	1525A/G, 1595T/C	100 Chinese patients with IVDD (31–81 years old), 100 controls (34–70 years old)	GG genotype of 1525A/G and CC genotype of 1595T/C associated with increased risk of IVDD and more severe forms of IVDD (grade IV)
Tan et al. (25)	Death receptor 4 (DR4)	rs4871857: C626G	296 Chinese Han patients with IVDD (mean age 48.42 years old), 208 controls (mean age 47.90 years old)	Mutant G allele OR = 1.958 for developing IVDD; GG and GC genotypes associated with more severe forms of IVDD
Williams et al. (54)	Growth differentiation factor 5 (GDF5)	rs143383	Meta-analysis including 5295 Northern European women (19–90 years old)	T allele OR = 1.72 for disc space narrowing and osteophyte production
Mu et al. (52)	Growth differentiation factor 5 (GDF5)	rs143383	892 Chinese male soldiers: 305 cases (mean age 21.94 years old), 587 controls (mean age 22.09 years old)	T allele OR = 2.115 for low back pain
Han et al. (55)	Vascular endothelial growth factor (VEGF)	-2578C/A, -634CC	102 Young Koreans with IVDD (mean age 23.6 years old), 139 controls (mean age 23.4 years old)	SNPs -2568CA or AA genotype, -634CC genotype OR = 21 for developing IVDD
Williams et al. (56)	Parkin	rs926849	Meta-analysis of 4600 Northern Europeans (18–85 years old)	Mutant C allele associated with reduced risk of IVDD
Rajasekaran et al. (34)	Cyclooxygenase 2 (COX2)	rs5277, rs5275	308 Indian male patients with mild Total Disc Degenerative Score (TDDS, mean 29.6 years old), 387 Indian male patients with severe TDDS (mean age 31.7 years old)	SNPs rs5277 and rs5275 significantly associated with IVDD
Gruber et al. (57)	Catechol-*O*-methyltransferase (COMT)	rs165656, rs4633, rs2095019, rs4708592	40 Patients with disc degeneration	SNPs rs165656, rs4633, rs2095019, and rs4708592 significantly associated with IVDD

**Table 2:** **Summary of proteins influenced by changes due to SNPs in their respective genes**.

System	Protein
Structural	Collagen I (20, 27, 28)
	Collagen IX (20, 29–33)
	Collagen XI (4, 33, 34)
	Aggrecan (24, 43–46)
Structural support	Carbohydrate sulfotransferase (47)
	Vitamin D receptor (12, 20, 48–51)
Cytokines	Interleukin-1a (35, 36)
	Interleukin-6 (13, 36, 37)
Extracellular matrix-degrading enzymes	Matrix metalloproteinase (MMP)-1 (58)
	MMP-2 (38, 39)
	MMP-3 (12, 40)
	MMP-9 (11)
	MMP-14 (41)
	A disintegrin and metalloproteinase with thrombospondin motif (ADAMTS)-4 (42)
	ADAMTS-5 (34)
Apoptotic factors	TNF (tumor necrosis factor)-related apoptosis-inducing ligand (TRAIL) (53)
	Death receptor 4 (25)
	Caspase-9 (1, 52)
	Parkin (56)
Growth factors	Growth differentiation factor 5 (52, 54)
	Vascular endothelial growth factor (55)
Pain mediators	Cyclooxygenase 2 (34)
	Catechol-*O*-methyltransferase (57)

### Collagens

Collagen is the most abundant protein found in the human body, with 28 different types. Throughout the body, the various collagen types are found in the ECM and have different structural support roles. Structurally, collagen fibers are composed of three polypeptide chains, referred to as α chains, that form one or more triple-helixes along their rod-shaped structure (59). When referring to the gene that produces a specific collagen type, the gene name and subunit name are given (e.g., collagen type IX alpha 2, *COL9A2*). The collagen types of interest to us are the ones found within IVDs: collagen I, II, IX, and XI.

The AF consists primarily of collagen I, a fibril-forming collagen. Fibrillar collagens – I, II, and III – are essential in defining the molecular and mechanical properties of a particular tissue (59). In the AF, collagen I is responsible for maintaining the tensile strength to withstand spinal compression, hydrostatic pressure, and keeping the NP contained. Collagen II is the primary collagen of the NP and is found as a loosely connected network (3). Various minor collagens such as collagen IX play an important supporting role in forming cross-links between different types of collagen, increasing structural strength. Collagen XI, although found in small amounts, is important in structural support of collagen II as well as forming connections between proteoglycans and collagen (5, 8). Considering the integral role of collagen in maintaining the structural integrity of the IVD, genetic polymorphisms affecting the function or abundance of collagen can predispose a patient to IVDD.

#### Collagen I

Collagen I, although found in both the NP and AF, is much more abundant within the AF of the IVD. Collagen I is made up of a helix consisting of two α1 chains, encoded by the collagen type I alpha 1 gene, *COL1A1*, and one α2 chain encoded by the collagen type I alpha 2 gene *COL1A2* (7). *COL1A1* contains a particular polymorphism that may be involved with increased risk of IVDD. Three noteworthy studies have established an association between the *COL1A1* Sp1-binding site SNP and IVDD (20, 27, 28). This particular SNP is a G to T substitution at position +1245, which is found within the first intron of the *COL1A1* gene (60). The change in nucleotides reportedly increases levels of *COL1A1* messenger RNA expression and subsequently COL1A1 protein expression (27). Investigators have hypothesized that the SNP leads to disequilibrium between COL1A1 and COL1A2 protein expression leading to instability of the collagen fibers (27, 28). Pluijm et al. examined 966 elderly (>65 years) Dutch individuals and reported that patients with the TT genotype had a 3.6-fold increased susceptibility to IVDD than patients with the GT or GG genotypes (27). The following year, Tilkeridis et al. examined the frequency of the Sp1-binding site polymorphism in 24 young Greek military recruits (28). The study reported that 33.3% of the patients with IVDD had the TT genotype while none of the control subjects did. Furthermore, the study indicated that 66.7% of the IVDD patients had the GT genotype while only 41.7% of the controls did. More recently, a 2015 study by Toktas et al. found that patients homozygous for the risk allele T had a significantly lower average Pfirrmann score (17.63) than patients without the allele (average score, 21.88) (20). They found a similar relationship between patients heterozygous for the allele compared with control patients. This study suggests that the *COL1A1* Sp1 polymorphism may not only be associated with an increased risk of developing IVDD but also associated with more severe forms of degeneration.

#### Collagen IX

Collagen IX is composed of three unique polypeptides, such as α1, α2, and α3, which are encoded by genes collagen type 9 alpha 1 (*COL9A1*), collagen type 9 alpha 2 (*COL9A2*), and collagen type 9 alpha 3 (*COL9A3*), respectively (20). Collagen IX is thought to play a significant role in connecting various types of collagens together, particularly collagen II (8, 59). Various studies have found SNPs located on either *COL9A2* or *COL9A3* that may be associated with increased risk of IVDD.

Annunen et al. examined 157 unrelated Finnish subjects with IVDD-induced sciatica (29). The study characterized a *COL9A2* polymorphism named Trp2, which caused a substitution of Gln or Arg for Trp in the collagen molecule. This substitution is particularly interesting because there are no naturally occurring Trp residues in collagen because the *COL9* gene does not encode for the amino acid Trp. The statistical analysis showed that patients with the allele coding for Trp were at a 4.5-fold increased risk of developing IVDD than those without the allele (29). Their population analysis found that 6 of the 157 individuals with IVDD had the Trp allele while none of the 174 controls did. A few other investigators have attempted to establish a connection between the Trp2 allele and IVDD but failed. For instance, Toktas et al. (20), Kales et al. (30), and Zhang et al. (31) did not find a correlation between *COL9A2* polymorphisms and IVDD.

A common SNP that has been studied in *COL9A3* is Trp3. This SNP is similar to the one found in *COL9A2*; it is an Arg103 to Trp substitution. Paassilta et al. studied the occurrence of the Trp3 allele in 171 Finnish subjects (32). The statistical analysis showed that patients who had a copy of the Trp3 allele were at a 2.7-fold increased risk of developing IVDD compared with patients who did not have the allele. Evidence for the association between the Trp3 allele and IVDD grew with a 2006 study by Solovieva et al. (33). They examined 135 middle-aged Finnish men and found that patients who carried the Trp3 risk allele in the absence of the IL-1 βT^3954^ SNP allele were at a 7.0-fold increased risk of a dark NP on MRI. These men had an overall 8.0-fold increased risk of degenerative changes in the spine. Although this study qualified the association between Trp3 and IVDD as dependent on the absence of the IL-1 βT^3954^ SNP allele, it nonetheless established a connection between the two (33). More recently, a 2015 study by Toktas et al. established a connection between the Trp3 allele and increased severity of disc degeneration (20). The study showed that of the five cases with Trp3 alleles, the heterozygous patients with the allele had a significantly lower average Pfirrmann score (19.40) compared with the wild-type patients without the allele (average score, 21.07). This finding suggests that not only is the Trp3 allele associated with an increased risk of developing IVDD but also associated with more severe forms of degeneration.

#### Collagen XI

Collagen XI has a similar structure to collagen IX in that it is a heterotrimer. The three chains, such as α1, α2, and α3, are coded by collagen type XI alpha 1 (*COL11A1*), collagen type XI alpha 2 (*COL11A2*), and collagen type II alpha 1 (*COL2A1*), respectively (5). Collagen XI is found in both the AF and NP of IVDs and has an important role in connections between the different collagen molecules, particularly collagen II, as well as connections between proteoglycans and collagen (5, 8).

Solovieva et al. showed a relationship between a G to A substitution SNP within intron 9 of *COL11A2* and disc bulging (33). Patients who were carriers of the SNP allele had a 2.1-fold increased risk of disc bulging compared with patients who did not have the allele. The study also noted a 1.6-fold increased risk of signs of disc degeneration, but the SD was too large to be statistically significant. Nonetheless, it is worth noting that the G to An SNP of *COL11A2* was related to change associated with disc degeneration. A 2009 study by Videman et al. documented five different polymorphisms in collagen XI genes that were significantly associated with signs of disc degeneration such as reduced disc signal and disc bulging (4). This particular large-scale study enrolled 588 Finnish male twins ranging from 35 to 70 years of age. The rs2072915, rs9277933, and rs2076311 SNPs of *COL11A2* were significantly associated with reduced disc signal on MRI, whereas the rs1337185 and rs1463035 polymorphisms of *COL11A1* were significantly associated with increased risk of disc bulging. A 2015 study by Rajasekaran et al. supported these findings (34). The study revealed the rs1337185 SNP of *COL11A1* was associated with a 1.55-fold increased risk of developing IVDD. Research suggests that SNPs in both *COL11A2* and *COL11A1* could predispose an individual to an increased risk of developing IVDD.

### Cytokines

Cytokines, such as IL-6, IL-1a, IL-1b, and tumor necrosis factor (TNF)-α, are some of the key pro-inflammatory mediators that are found and released at sites of tissue injury. IL-1 is naturally found within the IVD and is responsible for indirectly degrading ECM components through the production of degradative enzymes, upregulation of other cytokines, and preventing the production of ECM components (5). IL-1 has three different subtypes: IL-1a, IL-1b, and IL-1RN. The alpha and beta subtypes are pro-inflammatory, whereas IL-1RN is anti-inflammatory (7). Within the disc, a delicate homeostasis between the pro-inflammatory and anti-inflammatory subtypes exists that is easily disturbed by trauma to the spine and genetic polymorphisms.

A common SNP of interleukin 1 alpha (*IL1A*) was significantly associated with IVDD in a 2007 study by Virtanen et al. who examined 150 Finnish men (35). The SNP is an -889C/T substitution where the T allele is the risk allele. Patients in the study with the TT genotype were at a 7.87-fold increased risk of developing IVDD compared with patients with the CC genotype. These findings were supported by a 2012 study by Eskola et al. of Danish adolescents (36). The study found a 2.82-fold increased risk of developing IVDD among girls who were carriers of the T allele compared with the controls. The study also described the polymorphism as increasing IL-1a expression, and thus furthering its function as a cartilage destroyer (36). These two studies, along with a few others, established the -889C/T SNP of *IL1A* as a genetic risk factor for IVDD (7, 35, 36).

Interleukin-6 is an important mediator of inflammation and having involvement with lumbar disc herniation (36). Despite this information, the exact role of IL-6 in disc degeneration is not fully known (5). Noponen-Hietala et al. documented an SNP in the interleukin 6 gene (*IL6*) that was significantly associated with IVDD (37). A 15T/A substitution was located within exon 5 of *IL6*. Statistical analysis showed that patients with the AA or AT genotypes were at a 4.4-fold increased risk of IVDD than patients with the TT genotype. The study documented that the 15T/A SNP results in an exon 5 amino acid substitution that replaces Asp with Glu. The researchers hypothesized that this polymorphism led to disequilibrium of the pro-inflammatory cytokines and, therefore, accelerated inflammation (37).

Another SNP associated with *IL6* was described in a 2010 study by Eskola et al. (13). They identified SNP rs1800796, a 572G/C substitution, which was significantly associated with IVDD in Danish girls. The study found that female patients carrying the C allele were at a 6.71-fold increased risk of developing IVDD than those without the allele. This study did not find the same association in Danish boys (13). However, a 2012 study by Eskola et al. described two different SNPs of *IL6* that were found only in adolescent boys: rs1800797 and rs1800795. The G/A genotype (risk allele, G) of SNP rs1800797 was associated with a 0.27-fold decreased risk of developing IVDD, whereas the G/C genotype (risk allele, G) of SNP rs1800795 was associated with a 0.26-fold decreased risk of IVDD. Both polymorphisms were protective and potentially reduced the inflammatory tone of *IL6* (36). Overall, the research on *IL6* suggests that various polymorphisms may influence a patient’s genetic risk of IVDD; however, this effect may be limited to certain genders or populations.

### Matrix-Degrading Enzymes

Several types of matrix-degrading enzymes exist within the ECM of IVDs. Two of the major types of matrix-degrading enzymes that are involved in IVD degradation are MMPs and “a disintegrin and metalloproteinase with thrombospondin motif” (ADAMTS). The homeostasis of ongoing ECM turnover is managed by the balance between MMPs and tissue inhibitors of metalloproteinases (12). Various MMPs are responsible for degrading different substances. For example, collagen I, II, and III are primarily degraded by MMP-1, -8, and -13 – the collagenases, whereas denatured collagen is the target of MMP-2 and MMP-9 (59). It is important to remember that increased expression of MMPs leads to accelerated destruction of the ECM. ADAMTS are also referred to as aggrecanases because their primary function within the IVDs is to digest aggrecan (34). Similarly, an increase in expression of ADAMTS results in accelerated IVDD.

#### Matrix Metalloproteinase

Song et al. examined 691 southern Chinese people between the ages of 18 and 55 years and found an SNP at position -1607 in the promoter of the matrix metalloproteinase 1 gene (*MMP-1*) (58). The SNP was significantly associated with IVDD, and of the two alleles, D and G, the D allele was the risk allele. The statistical analysis revealed that patients carrying the D allele had a 1.41-fold increased risk of IVDD compared with those without the allele. Further analysis showed an even stronger connection in patients over the age of 40 years. In patients over the age of 40 years carrying the D allele, there was a 1.445-fold increased risk of developing IVDD. This study was particularly interesting because previous studies have shown the G allele of the -1607 SNP as increasing MMP-1 expression. The researchers hypothesized that expression of the D allele might lead to disequilibrium between the MMPs, and thus, greater degradation of the AF and NP (58).

MMP-2, one of the two gelatinases, tends to target denatured collagen as its substrate (59). Dong et al. found that the -1306C/T polymorphism of the *MMP2* gene was a genetic risk factor for IVDD (38). The study examined 162 Chinese young adults with disc degeneration. The statistical analysis demonstrated that patients with the CC genotype had a 3.08-fold increased risk of developing IVDD than those with at least one T allele (CT or TT). The study also found that the CC genotype was associated with more severe forms of IVDD than the CT and TT genotypes. This study was exceptionally interesting because the SNP is a C to T substitution, where the T allele is the risk allele, and the C allele is the wild-type. The T allele reduces Sp1 transcription factor binding to the gene and thus reduces overall transcription. The polymorphism that leads to increased protein production is the most likely one associated with an increased genetic risk of IVDD; in this case, it happened to be the wild-type C allele (38). A later study in 2013 by Zhang et al. revealed a similar phenomenon in the -735C/T polymorphism of *MMP2* (39). The study found that patients with the TT or CT genotypes had a 0.413-fold reduced risk of developing IVDD, whereas patients carrying the CC genotype were at nearly a 2.5-fold increased risk of developing IVDD compared with patients with the TT genotype. Similar to the -1306C/T SNP, the T allele was associated with disrupting a Sp1-binding site (CCACC box) and reducing transcription, while the C allele was considered the wild-type and was associated with increased transcription (39). These studies reveal that multiple nearby Sp1-binding sites whose polymorphisms are connected to genetic risk of IVDD exist (38, 39).

MMP-9 is also a gelatinase with variable expression that has been linked to IVDD. A 2009 study by Sun et al. revealed a -1562C/T polymorphism that affected the protein expression of MMP-9 (11). Patients with the CT/TT genotypes were at a 2.14-fold increased risk of developing IVDD compared with patients with the CC genotype. The T allele is associated with increased MMP-9 expression, and thus an imbalance between MMPs and tissue inhibitors of metalloproteinases, leading to excessive degradation of the ECM (11).

MMP-3 is one of the three MMPs that are categorized as stromelysins (59). One of the main functions of stromelysins is to degrade proteoglycans, laminas, and other components of the IVD ECM as well as indirectly degrade the disc through activating other MMPs (40). Expression of MMP-3 has also been shown to rise in response to inflammation (51).

The most commonly studied SNP of *MMP3* is the 5A variant allele in the promoter region of the gene. A 2001 study by Takahashi et al. revealed that elderly patients who had the 5A/5A or 5A/6A genotype were at an increased risk of IVDD (40). However, the study did not find this association in younger patients. Yuan et al. investigated the same 5A polymorphism and found that patients who carried the shorter 5A allele were at a 1.96-fold increased risk of developing IVDD (12). More recently, Zawilla et al. found that the 5A allele was associated with a 2.5-fold greater risk of developing IVDD (51). The study also found a link between the 5A allele and increased severity of degradation. An abundance of evidence suggests that the shorter 5A polymorphism of *MMP3* is linked to an increased genetic risk of IVDD (51).

MMP-14 is a membrane-anchored MMP that is found at the cell surface and is involved in degrading small fragments of collagen and activating *MMP2* (41, 59). Researchers have hypothesized that overexpression of MMP-14 leads to overall disc degradation mainly through the activation of *MMP2* (41). In a 2015 study by Zhang, the -378T/C SNP in *MMP14* was a genetic risk factor associated with IVDD (41). Patients with the TT genotype had a 1.59-fold increased risk of IVDD compared with patients with the CC genotype.

Considering all the various SNPs associated with MMPs and their influence on patients’ risk of developing IVDD, protein expression levels are a delicate and important aspect of ECM maintenance of IVDs. It is possible that genetic manipulation of MMPs is a significant factor in the etiology behind IVDD. Furthermore, MMPs are strong candidates for therapeutic options for mitigating or reversing IVD degradation.

#### A Disintegrin and Metalloproteinase with Thrombospondin Motif

A disintegrin and metalloproteinase with thrombospondin motif are enzymes that play a central role in disc degeneration *via* aggrecan turnover (42). In particular, ADAMTS-4 and ADAMTS-5 are found at the site of disc degeneration. Various genetic polymorphisms in the *ADAMTS* family of genes are linked to the risk of IVDD. Liu et al. were the first to investigate a polymorphism in *ADAMTS4* (42). They found that SNP rs4233367, an 1877C/T substitution, was associated with a reduced risk of IVDD. Patients with the TT genotype were at a 0.21-fold reduced risk of developing IVDD compared with those with the CC genotype. This strong connection suggests that *ADAMTS4* plays an important part in proteoglycan degradation within the IVD. Rajasekaran et al. investigated SNP rs162509 in *ADAMTS5* and found that the risk allele was associated with a 1.281-fold increased risk of developing IVDD (34). Although this relationship is small, it supports the notion that ADAMTS proteins are essential for the maintenance of healthy, hydrated discs.

### Aggrecan

Aggrecan is the most plentiful proteoglycan found within the IVD, and its primary function is to retain water. The core protein of aggrecan contains a large number of chondroitin sulfate and keratin sulfate chains that facilitate its ability to create an osmotic gradient. Furthermore, aggrecan binds to negatively charged glycosaminoglycans to increase the hydrostatic pressure of the NP (5, 7). One of the most investigated polymorphisms of aggrecan is the variable nucleotide tandem repeat (VNTR) in the chondroitin sulfate-1 encoding domain of the aggrecan gene (*ACAN*) (45). The chondroitin sulfate encoding allele has VNTRs ranging from 13 to 33 nucleotides, with the most common number being 26, 27, or 28 repeats. As aggrecan water-retention abilities are heavily reliant on the number and size of chondroitin sulfate chains, it makes sense that a reduced number of repeats would impair the ability of aggrecan to retain water (24, 43–46). One of the earliest studies published on this topic was in 1999 by Kawaguchi et al. (43). The study found patients with 18 or 21 repeats in the chondroitin sulfate encoding domain were at an increased risk of multilevel disc degeneration as well as more severe forms of degeneration when compared with patients with longer alleles. A 2010 study by Eser et al. supported these results (24). They found that patients with short alleles, consisting of VNTRs of A13 to A26, were at an increased risk of severe disc degeneration compared with those with longer VNTRs in their alleles. The study also found that patients with short, A13 to A26, or normal, A27, were at an increased risk of multilevel disc degeneration. These findings were further supported by a 2012 study by Xu et al. who found that patients with less than 23 VNTRs were at a 1.95-fold increased risk of IVDD compared with those with more than 23 repeats (44). The study also found that patients with less than 25 repeats were at a 1.85-fold increased risk of IVDD compared with those with more than 25 repeats. This study helped establish the dose-dependent nature of the VNTRs of the aggrecan gene. The risk associated with VNTRs seems to follow a continuous scale, as opposed to a Boolean, or “cut-off” pattern (44). A 2013 meta-analysis by Gu et al. revealed that patients with shorter alleles, A13 to A25, were at a 1.54-fold increased risk of IVDD compared with those with either normal, A26 to A27, or longer alleles, A28 to A32. This relationship was found to be even stronger in patients of Asian descent, who were at a 1.65-fold increased risk of IVDD (45). This study helped solidify the notion that shorter VNTRs are not only associated with increased risk of IVDD but also suggest that the magnitude of the effect may be associated with race.

In 2007, Solovieva et al. investigated the VNTRs for the aggrecan gene in 132 middle-aged Finnish men (46). Their analysis found that the A26 allele was associated with an increased risk of the patient’s NP to be dark on an MRI scan, which is an indication of IVDD. The study also found that patients with A26/A26 genotype were at a 2.77-fold increased risk of a dark NP compared with patients who had longer or shorter VNTRs. This study is unique and did not follow the same trends as the previously mentioned studies. In previous studies, A26 was either considered within normal/typical range or even long (24, 43–45). This 2007 study helped support the notion that the effects of VNTRs may also be influenced by the race or ethnicity of the patient.

Various other genes that affect the aggrecan water-retention abilities or aggrecan expression have also been investigated. Carbohydrate sulfotransferase 3 (CHST-3) is an enzyme that is involved in sulfation of the aggrecan side-chains and is coded by *CHST3*. This function makes CHST-3 an important and indirect contributor to disc hydration. Song et al. identified the SNP rs4148941 that produced the risk allele A (47). They found that the allele A variant of *CHST3* had improved binding with micro RNA sequence miR-513a-5p. Their statistical analysis showed that patients with the AA or AC genotypes were at a 1.48-fold increased risk of developing IVDD. Further analysis revealed that the A allele was associated with reduced expression of the *CHST3* messenger RNA within the IVDs, suggesting reduced expression of CHST-3 protein (47). Overall, the study established the SNP of *CHST3* as a genetic risk factor for IVDD.

### Vitamin D Receptor

**Figure 3:**
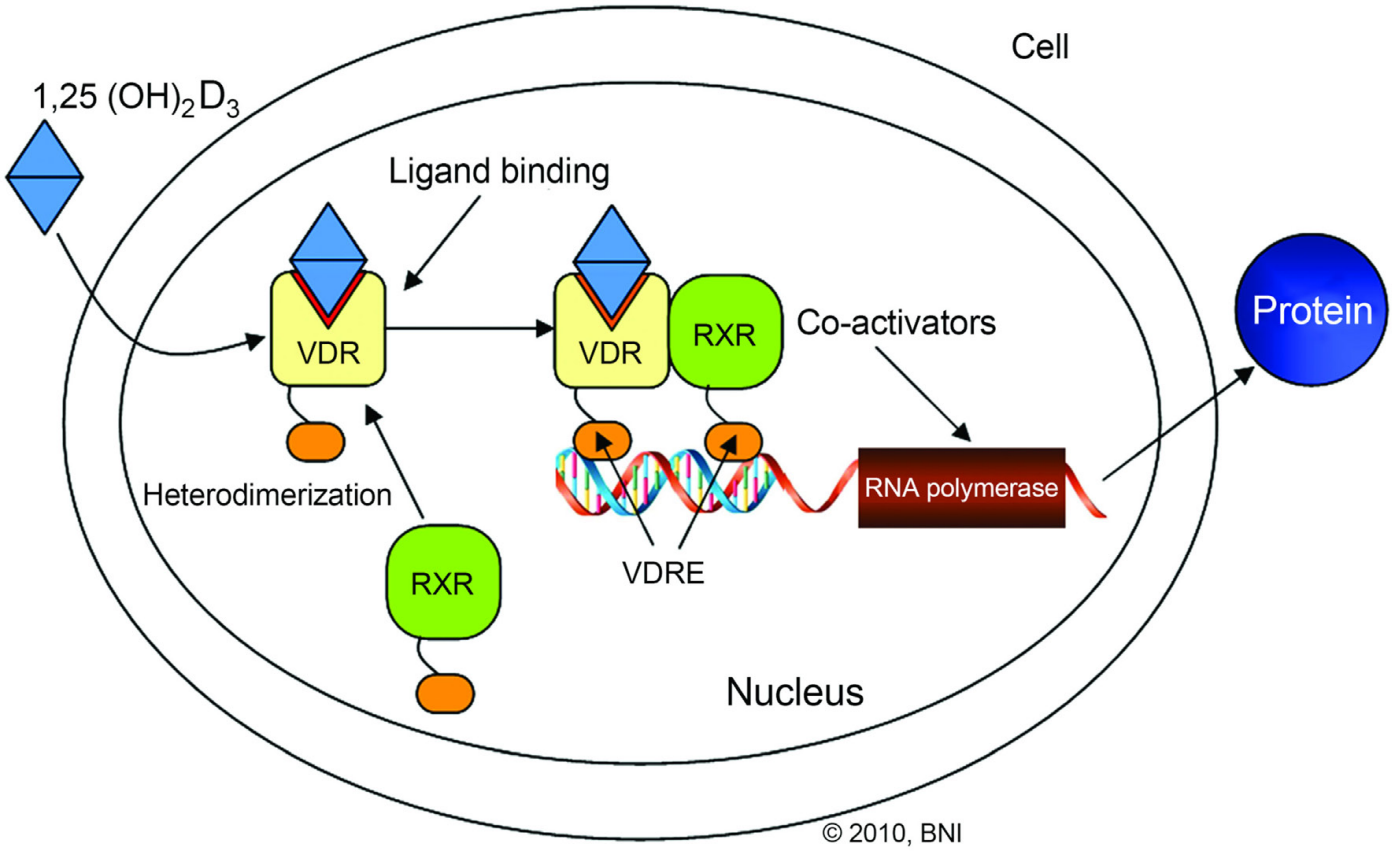
**Illustration outlining the vitamin D receptor (VDR) signaling pathway**. VDR/retinoid X receptor (RXR) interaction with vitamin D response element (VDRE) stimulates gene transcription. Used with permission from Barrow Neurological Institute, Phoenix, AZ, USA.

The VDR is a nuclear receptor for a vitamin D metabolite, 1a,25-dihydroxyvitamin D3 (Figure 3). Previous studies have shown that *VDR* polymorphisms are associated with various bone disorders including osteoarthritis, osteoporosis, and cardiovascular disease (7, 44). VDR function in IVDs is hypothesized to be through an indirect pathway for chondrocyte proliferation and the effect of chondrocytes on proteoglycans (12). Over the past two decades, various polymorphisms affecting the expression and function of VDRs in IVDD have been identified. These SNPs include FokI (rs2228570), TaqI (rs731236), and ApaI (rs7975232) (12, 20, 24, 44, 48, 49, 51, 61, 62).

The FokI polymorphism of *VDR* is a C to T substitution found in exon 2 (49). This SNP leads to altered protein size, and subsequently, altered function. Research has shown that the shorter polypeptide of VDR is associated with the wild-type C variant. The F allele has a higher affinity for transcription factor II B. The wild-type alleles lead to normal functioning *VDR*, while the T substitution (risk allele f) is associated with reduced function (62). A 1998 study by Videman et al. of Finnish twins found that the ff genotype was associated with 9.3% reduced signal intensity within the T6–S1 region on an MRI compared with the FF genotype (48). They also found that the Ff genotype was associated with 4.3% reduced signal intensity within the same region. These results were supported by a 2010 study by Eser who found that the FF genotype was associated with milder grades of degradation (grades I and II), whereas the ff genotype was associated with more severe grades (grades III and IV) (24). The FokI SNP was not only associated with an increased severity of IVDD but also increased the risk of developing IVDD. Vieira et al. found that the T allele was associated with a 1.58-fold increased risk of developing IVDD compared with the C allele (49). These results were further supported by a recent 2016 study by Zhao et al. They found that Hispanic patients with the ff or Ff genotype (TT or TC alleles) were at a 1.742-fold higher risk of developing IVDD, whereas Asian patients with similar genotypes had a 1.293-fold increased risk (62). The data on the FokI SNP suggest that it is a genetic risk factor not only for IVDD but also for the severity of IVDD. Furthermore, these data suggest that the FokI polymorphism manifests differently in patients based on race or ethnicity.

Another significant SNP of *VDR* that has been the target of the most investigation among *VDR* polymorphisms is the TaqI variant (44). Interestingly, TaqI is a silent mutation in exon 9 of the *VDR* gene, yet it has a profound effect on a patient’s genetic risk of developing IVDD (50). One of the earliest studies of the TaqI polymorphism was the 1998 study by Videman et al. (48). They found that the patients with the tt genotype displayed 12.6% reduced signal intensities in the T6–12 range on MRI compared with patients with the TT genotype. These findings were supported by a 2002 study by Kawaguchi et al. who investigated the incidence of the TaqI SNP in Japanese young adults (50). The study found that patients with the Tt genotype were at an increased risk of multilevel IVDD and more severe forms of degeneration. The study was unable to establish the same connection for the tt genotype because none of the subjects had the tt genotype. In 2010, Eser et al. found that patients with the TT genotype displayed significantly milder forms of IVDD than patients with the tt genotype (24). A study in 2015 by Toktas et al. supported the association of the TaqI SNP with increased severity of disc degeneration. They found that patients with the homozygous tt genotype had an average Pfirrmann score of 18.45, which was significantly lower than in those with wild-type genotypes (average score, 22.15) (20). The findings from these studies suggest that the TaqI SNP of *VDR* is associated with both increased risk of developing IVDD and severity of IVDD.

Another common polymorphism of *VDR* that has received much attention is the ApaI SNP. The ApaI SNP maps to intron 8 of *VDR* and is associated with increased risk of IVDD (50). Yuan et al. found that the risk allele of the ApaI SNP was associated with a 1.70-fold increased risk of developing IVDD (12). These findings are supported by a 2013 study by Zawilla et al. who found that the mutant T allele of *VDR* was associated with a 3.1-fold increased risk of developing IVDD (51). They also found that the mutant T allele was significantly associated with increased severity of IVDD. Although the ApaI polymorphism is associated with both severity and risk of developing IVDD, the exact mechanism and its impact on the VDR protein has not been thoroughly investigated (12, 50, 51). Despite this, ApaI is a well-established genetic risk factor of IVDD.

### Apoptosis

Studies regarding the molecular mechanisms of IVDD have established that degenerated discs display much higher rates of apoptosis, programed cell death (3, 5). Although the exact cascade of molecules involved in apoptosis of IVD cells remains under investigation, there are a few significant genes whose polymorphisms have been associated with increased risk of IVDD. Among these are caspase-9 (*CASP9*), TNF-related apoptosis-inducing ligand (*TRAIL*), and death receptor-4, *DR4*, also known as *TRAIL* receptor 1 (*TRAILR1*) (1, 10).

Caspase-9 is an important activator of the intrinsic pathway of apoptosis. Its expression levels within the IVD have been reported to increase during disc degeneration (1). The first study to report on *CASP9* polymorphisms and their relationship to IVDD was a 2011 study by Guo et al. (1). The study investigated two SNPs, rs4645978 (-1263A/G) and rs4645981 (-712C/T). They analyzed data from 154 patients with IVDD and found that the mutant GG genotype was associated with a 2.760-fold increase in the risk of IVDD compared with the AA genotype (1). Mu et al. investigated the same polymorphism, -1263A/G, and found that the G allele was associated with a 2.059-fold increase in the risk of developing lower back pain compared with the A allele (52). These studies suggest that SNPs affecting the expression and function of apoptosis factors may be another way in which genetic factors influence the progression of IVDD.

DR4 and DR5 are both receptors that bind to TRAIL and induce apoptosis within the target cell. Recent studies have shown that the TRAIL/DR4/DR5 system is important in mediating apoptosis within IVDs (10). Polymorphisms that influence the function and expression of either *TRAIL* or *DR4* can significantly impact the rate of apoptosis occurring within IVDs. Xu et al. identified two polymorphisms of *TRAIL* within the 3′-untranslated region, such as 1525A/G and 1595T/C, which are associated with IVDD (53). The mutant GG genotype at the 1525 locus and the mutant CC genotype of the 1595 locus were associated with increased risk of IVDD. The investigation found that both the GG1525 and CC1595 genotypes were associated with reduced TRAIL expression within the cells as well as more severe forms of IVDD (grade IV). Although reduced TRAIL expression has already been established in IVDD, the underlying pathophysiology remains under investigation (53).

The *TRAIL/DR4/DR5* system is also affected by polymorphisms in *DR4*. Tan et al. found that degenerating IVD cells had increased expression of DR4 (25). They investigated a Chinese Han population and found that SNP rs4871857 (626C/G) in exon 4 of *DR4* was associated with IVDD. Patients with the mutant G allele were at a 1.958-fold increased risk of developing IVDD. Furthermore, the GG and CG genotypes were associated with more severe grades of IVDD (25). The findings on *TRAIL* and *DR4* revealed another aspect of IVDD that may be controlled by genetic factors.

### Growth Factors

Growth differentiation factor 5 (GDF5) is part of the transforming growth factor-β superfamily involved in bone, ligament, and soft tissue development (52, 54). Increased GDF5 expression is linked to increased collagen II and aggrecan production in human IVDs (63, 64). An investigation of polymorphisms in *GDF5* revealed that its variable expression and function are linked to osteoarthritis. Williams et al. investigated SNP rs143383 (a T to C substitution at position 104) located within the promoter region of the *GDF5* gene. Their analysis showed that the T allele was associated with 1.72-fold increased risk of disc space narrowing and osteophyte production in women (54). These findings are supported by a 2013 study by Mu et al. who investigated the same SNP (52). They found that the T allele of *GDF5* was associated with a 2.115-fold increased risk of lower back pain. Although the study revealed an association between the T allele and lower back pain, the findings still suggest the involvement of *GDF5* polymorphisms in IVDD.

Similar studies have investigated the influence of vascular endothelial growth factor (*VEGF*) gene polymorphisms and their link to IVDD (55). IVDs are some of the largest avascular structures within the human body. Consequently, they rely on small capillaries extending from the lumbar artery to help remove metabolic waste (5). One of the main features of a severely degenerated disc is neovascularization penetrating the AF, hence, the interest in VEGF, a key mediator of angiogenesis (55). Han et al. found that when a patient possessed multiple *VEGF* SNPs, there was a significant association with IVDD (55). For example, a patient with the genotype of -2578CA or AA, combined with -634CC genotype, was at a 21-fold increased risk of IVDD. With limited data, it is difficult to conclude with certainty that *VEGF* SNPs are associated with IVDD; however, Han et al. (55) have helped establish the preliminary data to warrant further investigation into *VEGF* polymorphisms.

### Ubiquitin-Mediated Degradation

E3 ubiquitin-protein ligase is a multiprotein complex that functions in an ubiquitin–proteasome pathway, marking proteins for degradation. A key protein in this complex named Parkin is expressed in various organs and skeletal muscles. Parkin is coded by *PARK2*, which was recently associated with IVDD (8, 56). In a 2013 study of 4600 Northern Europeans, Williams et al. reported that the rs926849 SNP is a T to C substitution found within an intron of *PARK2* (56). Their statistical analysis revealed that the C allele was significantly associated with reduced risk of IVDD, suggesting that the C allele was protective. The underlying mechanism of how the C allele influences the expression of *PARK2* and the subsequent pathology remains under investigation (56). Nonetheless, this study adds another component to the etiology of IVDD as well as highlighting the complexity and continued discoveries associated with IVDD.

### Cyclooxygenase

Cyclooxygenase 2 is an essential enzyme that is involved in the production of various prostaglandins and thromboxanes. The cyclooxygenase 2 gene *COX2* and its products participate in multiple pathways including inflammation and pain (8, 34, 65). In 2015, Rajasekaran et al. identified two SNPs, such as rs5277 and rs5275, in *COX2* that are significantly associated with severe IVDD (34).

### Catechol-*O*-Methyltransferase

Catechol-*O*-methyltransferase is an enzyme that is involved in the degradation and processing of catechol neurotransmitters such as dopamine. Previous clinical studies showed a relationship between certain polymorphisms in the catechol-*O*-methyltransferase (*COMT)* gene and pain. The IVDD researchers believed that variable catechol-*O*-methyltransferase expression led to increased pain in IVDD. Gruber et al. identified four *COMT* SNPs, such as rs4633, rs165656, rs2095019, and rs4708592, significantly associated with IVDD (57). Their findings supported results that were previously published regarding the association of rs4633 and IVDD. Although rs165656 has previously been associated with mental retardation, Gruber et al. were the first to show its significant association with IVDD (57). The rs2095019 and rs4708592 polymorphs are novel SNPs that have not been reported previously (57). The study is a strong indicator of the complexity of the acute and chronic changes that occur with IVDD as well as highlighting the ongoing research that has revealed new aspects of its etiology.

### Personalized Medicine

The ultimate goal in reviewing the medical literature about the genetic polymorphisms associated with IVDD is to provide patients with personalized and targeted therapeutics. When a patient enters a clinic with lower back pain and degenerative disc disease is suspected, an MRI can provide a conclusive diagnosis. To provide targeted treatment for the specific patient, the physician must understand the patient’s unique molecular profile. Through gene sequencing and screening for SNPs, physicians can obtain a better understanding of the imbalances that led to the patient’s disc degeneration. Some patients may primarily have imbalances with ECM degrading enzymes, whereas others may have overexpression of proapoptotic factors. With this information, unique to each patient, specific therapies can be selected to provide the best long-term outcome.

## Conclusion

Despite continued research, the etiology and pathophysiology underlying IVDD remain poorly understood (34). Nonetheless, a significant shift in the understanding of IVDD has occurred over the past two decades, and we now understand that roughly 75% of the etiology behind IVDD is genetic (2, 6). One of the crucial techniques that have helped researchers to realize this understanding is the advent of large-scale DNA arrays and computational analysis software to analyze polymorphisms quickly (34). These techniques have helped bring to light new proteins and associations within systems that were previously thought not to be linked to IVDD. With a better understanding of the pathophysiology of IVDD and improved technology for scanning entire genomes for SNPs than in the past, we expect to produce innovative, new therapeutic approaches.

Two important aspects of genetic polymorphisms that have come to light are variations in race and ethnicity. Some polymorphisms tend to have stronger, or even no effect, on certain races. For example, Hispanics with the FokI SNP of *VDR* were at a much higher risk of IVDD than their Asian counterparts. Furthermore, the same meta-analysis found that the FokI SNP was not associated with IVDD in people of Caucasian decent (62). These racial variations add a new aspect and complexity to the understanding of the genetic factors underlying IVDD.

With more than 20 unique polymorphisms associated with IVDD, the molecular changes in the associated proteins or pathology of the disc are not yet fully understood. In the coming years, research targeted toward fully understanding the protein changes due to the already identified SNPs is crucial. If we can fully understand the molecular changes involved in IVDD then creating targeted therapeutics based on genetic profiling becomes a possibility.

With improved understanding of the genetic variants associated with IVDD, and rapid genomic analysis available through next-generation genotype sequencing, the possibility of providing effective personalized medicine can become a reality (14, 15). This comprehensive literature review regarding the genetic variants associated with IVDD not only affords a better understanding of the molecular mechanisms behind IVDD but also allows physicians the possibility of providing targeted treatments. For instance, if an IVDD patient were identified to have genetic variants resulting in the overexpression of apoptotic factors, physicians would be able to refine their therapy and target the specific underlying IVDD-causing mechanism unique to that patient. Furthermore, genomic screening for the known variants associated with IVDD can help predict disease progression and severity. This knowledge can help provide more effective treatments personalized to the unique phenotypic presentation of the patient. Considering the majority of the etiology underlying IVDD is genetic, it is essential that researchers and clinicians have a keen understanding of this underlying etiology to optimize treatment (2). Consequently, DNA screening for the genetic variants explaining the pathophysiology of the patient’s IVDD should be the standard of care.

## Author Contributions

All authors made substantial contributions to the conception or design of the work.

## Conflict of Interest Statement

The authors declare that the research was conducted in the absence of any commercial or financial relationships that could be construed as a potential conflict of interest.

## Abbreviations

ADAMTS, a disintegrin and metalloproteinase with thrombospondin motif; AF, annulus fibrosis; ECM, extracellular matrix; GDF5, growth differentiation factor 5; IL, interleukin; IVD, intervertebral disc; IVDD, intervertebral disc degeneration; MMP, matrix metalloproteinase; MRI, magnetic resonance imaging; NP, nucleus pulposus; SNP, single-nucleotide polymorphism; VEGF, vascular endothelial growth factor; VNTR, variable nucleotide tandem repeat.
